# Distinct Annular Oligomers Captured along the Assembly and Disassembly Pathways of Transthyretin Amyloid Protofibrils

**DOI:** 10.1371/journal.pone.0044992

**Published:** 2012-09-12

**Authors:** Ricardo H. Pires, Árpád Karsai, Maria J. Saraiva, Ana M. Damas, Miklós S. Z. Kellermayer

**Affiliations:** 1 Department of Biophysics and Radiation Biology, Faculty of Medicine, Semmelweis University, Budapest, Hungary; 2 Institute for Molecular and Cell Biology, University of Porto, Porto, Portugal; 3 Department of Biophysics, University of Pécs, Pécs, Hungary; 4 Instituto de Ciências Biomédicas de Abel Salazar, University of Porto, Porto, Portugal; Swiss Federal Institute of Technology Zurich, Switzerland

## Abstract

**Background:**

Defects in protein folding may lead to severe degenerative diseases characterized by the appearance of amyloid fibril deposits. Cytotoxicity in amyloidoses has been linked to poration of the cell membrane that may involve interactions with amyloid intermediates of annular shape. Although annular oligomers have been detected in many amyloidogenic systems, their universality, function and molecular mechanisms of appearance are debated.

**Methodology/Principal Findings:**

We investigated with high-resolution *in situ* atomic force microscopy the assembly and disassembly of transthyretin (TTR) amyloid protofibrils formed of the native protein by pH shift. Annular oligomers were the first morphologically distinct intermediates observed in the TTR aggregation pathway. Morphological analysis suggests that they can assemble into a double-stack of octameric rings with a 16±2 nm diameter, and displaying the tendency to form linear structures. According to light scattering data coupled to AFM imaging, annular oligomers appeared to undergo a collapse type of structural transition into spheroid oligomers containing 8–16 monomers. Disassembly of TTR amyloid protofibrils also resulted in the rapid appearance of annular oligomers but with a morphology quite distinct from that observed in the assembly pathway.

**Conclusions/Significance:**

Our observations indicate that annular oligomers are key dynamic intermediates not only in the assembly but also in the disassembly of TTR protofibrils. The balance between annular and more compact forms of aggregation could be relevant for cytotoxicity in amyloidogenic disorders.

## Introduction

Severe degenerative diseases, such as Alzheimer’s and Parkinson’s diseases, type II diabetes mellitus, spongiform encephalopathy and a wide range of amyloidoses are caused by the appearance of misfolded proteins which become deposited in various tissues as amyloid fibrils and plaques [1]. However, the severity of the diseases does not necessarily correlate with the amount of amyloid deposits [2], and the common cellular and molecular mechanisms behind these disorders remain unclear. It has been proposed that, rather than mature fibrils, smaller soluble oligomeric amyloid intermediates are responsible for the cytotoxic effects [1]. In particular, cytotoxicity has been linked to an oligomeric arrangement with annular morphology. The pore-like structure of annular oligomers is suggestive of a cytotoxicity mechanism (similar to that employed by some pathogenic bacteria) via the formation of pernicious ion-channels [3]. Indeed, as observed in electrophysiological recordings [4], single-liposome leakage assays [5], and in vivo cell imaging [3] amyloid oligomers appear to form discrete ion channels in lipid membranes which are thought to perturb ion homeostasis, eventually leading to cell death. Furthermore, the occurrence of amyloid annular oligomers across a large range of amyloidogenic systems [6] and their presence in post-mortem tissues [7] suggest that they may be a common link in amyloidogenesis. Annular oligomers are not the only intermediates that appear during amyloid fibrillogenesis. Non-porous species such as spheroid oligomers and worm-like protofibrils have also been reported [8]. The structural relationship between the different intermediates and their contribution to cytotoxicity remain unclear.

In the present work we investigated the assembly and disassembly of transthyretin (TTR) amyloid protofibrils with high-resolution *in situ* atomic force microscopy (AFM). TTR is a tetrameric protein that plays an important role in the transport of retinol and thyroxin under physiological conditions [9], [10]. TTR is associated with two clinical forms of systemic amyloidosis, senile and hereditary [11], [12]. Typically, mutant forms of TTR result in amyloidogenesis [13], but it has been demonstrated that amyloid-like fibrils may form *in vitro* from wild-type (WT) TTR at low pH [14]. Recently we explored the structure of WT TTR protofibrils with AFM and documented structural changes evoked by mild acidification and pH shift [15]. Here we report the appearance of distinct annular oligomeric intermediates formed along both the assembly and disassembly pathways of TTR protofibrils formed in the acid-induced amyloidogenesis pathway. Our observations suggest that annular oligomers undergo morphological transitions into spheroid oligomers and protofibrils. We further demonstrate that protofibril state may be reverted to an annular oligomer configuration at near physiological conditions, which may be relevant for the dynamics of toxic structural transitions.

## Results

### Assembly of Annular Oligomers

**Figure 1:**
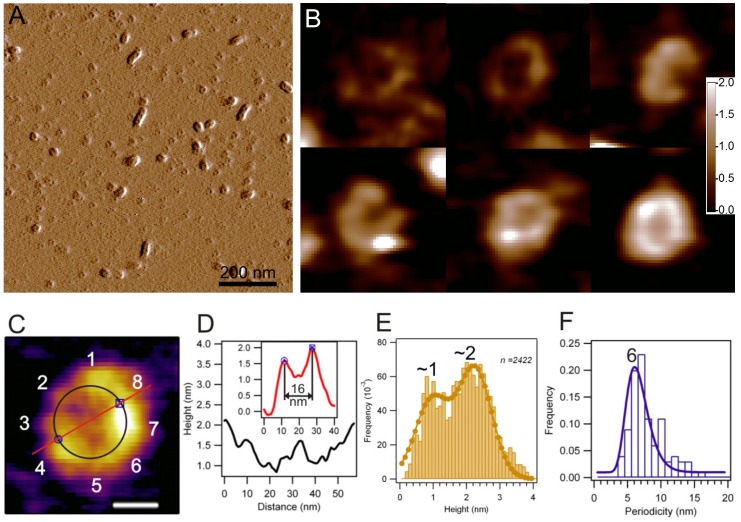
Annular oligomers along the TTR assembly pathway. A. 2×2 µm AFM scan displaying TTR oligomers some of which form short (<100 nm) linear structures. **B.** 500×500 nm AFM scan of the square area marked in **Figure 1** **.A**, indicating that the sample is almost exclusively populated by annular oligomers. In the center, annular oligomers are associated to form a linear aggregate. **C.** Height contrast image of an annular TTR oligomer. Octameric symmetry is indicated with numbers referring to component monomers. The circle and line mark the perimeter and diameter, respectively, across which topographical height data were measured and plotted. Scale bar, 10 nm. **D.** Topographical height profiles of the annular oligomer taken along its perimeter (black) and its diameter (inset, red). **E.** Distribution of the topographical height of annular oligomers. “*n*” represents the number of height data points for 57 annuli. **F.** Distribution of peak-to-peak periodicity along the annulus perimeter (*n* = 106; 33 annuli).

**Figure 2:**
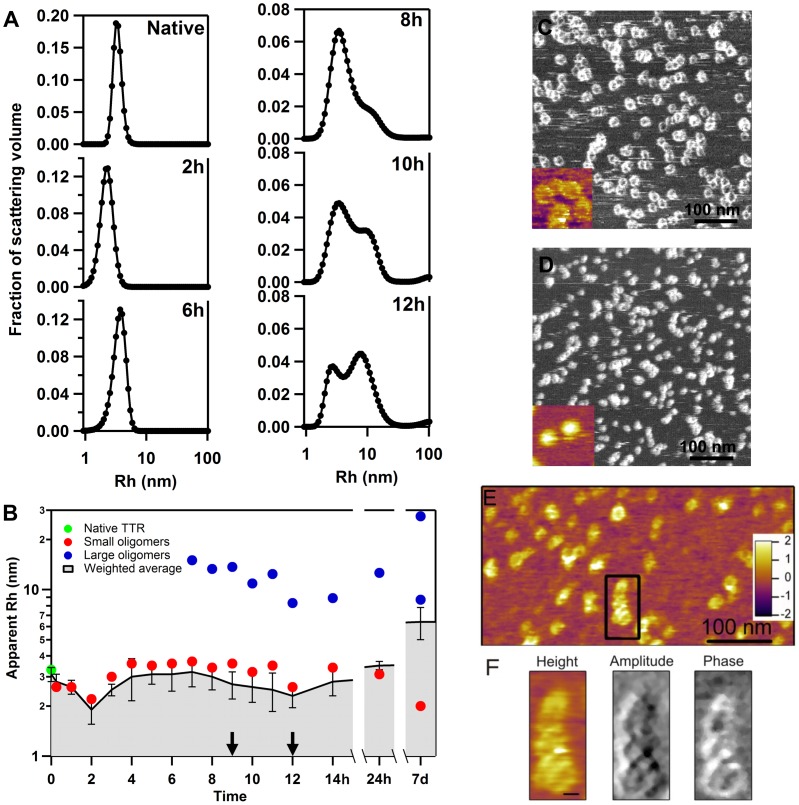
Formation and disappearance of annular oligomers. **A.** Dynamic light scattering spectra of native TTR and at given time points after the start of acidification where the emergence of a small population of larger particles follows a trend towards smaller apparent hydrodynamic radii (Rh_app_). **B.** Time course of the apparent size of the different populations during aggregation and their corresponding weighted average. The arrows indicate the times points where images shown in C and D were taken. **C & D.** AFM images (phase contrast) of particles taken at 9 and 12 h respectively and where annular oligomers (C) and spheroid (D) oligomers can be observed. The inset represents a 50×50 nm topography image of the corresponding samples (height scale up to 2.5 nm) **E.** Height-contrast AFM image of annular oligomers undergoing transitions. **E.** Magnified view of fusing annular oligomers indicated in *D*. Height, amplitude and phase contrast images (left to right) are shown. Scale bar, 10 nm.

Images of native WT TTR revealed particles with a distribution of height and molecular volume showing predominant peaks at 1.0 nm (±0.9 nm S.D., n = 204 particles) (Figure S2) and ∼25 nm^3^ (±138 nm S.D., n = 204 particles) (Figure S3), respectively. To evoke amyloidogenic structural transitions in WT TTR and a commitment towards the amyloidogenic pathway, the pH of the buffer solution was lowered to 3.6 [15], [16]. In the first hours of incubation the sample was nearly homogenously populated by particles with a topographical height of ∼0.9 nm (±0.3 nm S.D., n = 138 particles) and a molecular volume of ∼40 nm^3^ (±21 nm^3^ S.D., n = 138 particles). Between 9 and 24 hours of incubation annular structures appeared (Figures 1 and 2). These annular oligomers were most often isolated, but occasionally they lined up to form short elongated structures up to 60 nm in length (Figure 1A). Both the shape and the size of the annuli were uniform, and there was little deviation from circular geometry (Figs. 1B-C). The structure of annular TTR oligomers was investigated in AFM images (Figure 1C). Their diameter (Figs. 1C-D) was 16 nm (±2 nm S.D.; n = 57 particles). Their topographical height distributed across two peaks at ∼1 and ∼2 nm (Figure 1E), suggesting that annuli with 1 nm in height might stack on each other. We could resolve a topographic periodicity of 6 nm (±3 nm S.D.; n = 106; 33 particles, Figure 1F) along the annular perimeter (∼50 nm), indicating an octameric symmetry which could be clearly discerned in some particles such as the one shown in Figure 1C. Attempts to obtain higher resolution images by drying and scanning in air a sample containing annular oligomers were unsuccessful, since this resulted in the observation of spherical particles with a much higher topographical profile of approximately 10 nm, likely resulting from the drying procedure (Figure S4).

### Disappearance of Annular Oligomers Results in the Formation of Spheroid Oligomers and Protofibrils

**Figure 3:**
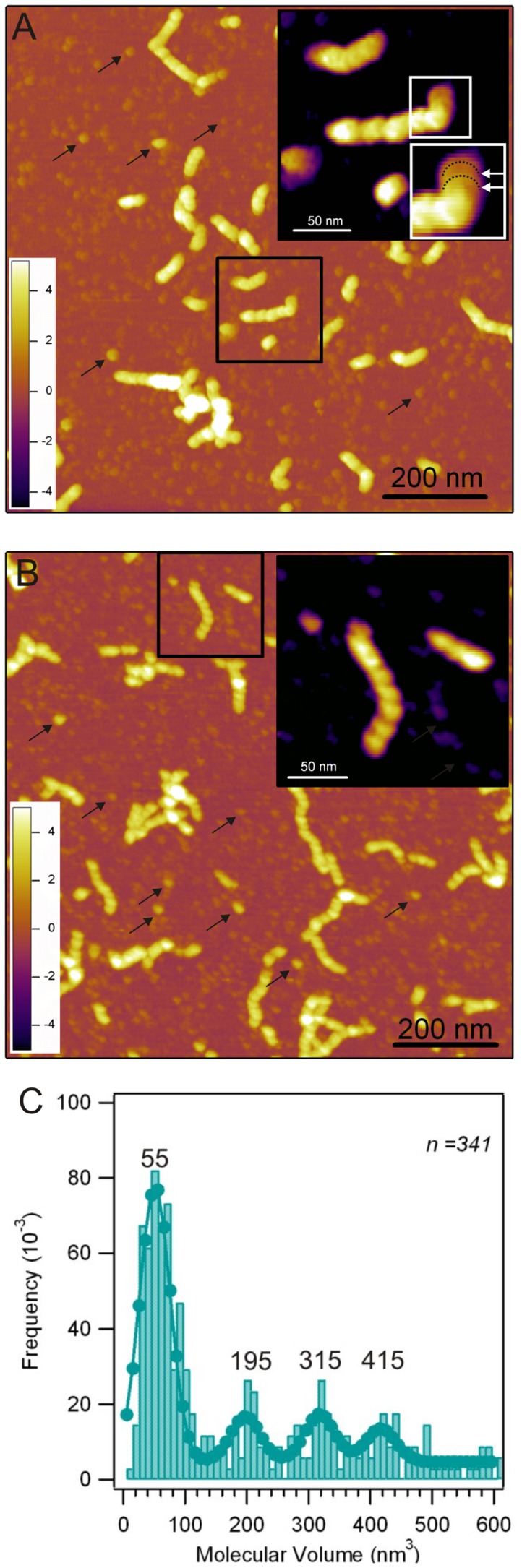
TTR spheroid oligomers and protofibrils. **A.** 1×1 µm^2^ AFM height contrast image of a mixed population of spheroid oligomers and short protofibrils. Black arrows point out examples of spheroid oligomers with various shapes and sizes. **Inset,** magnified view of a protofibril displaying a stack-like arrangement of flat, disc-shaped oligomers reminiscent of annular origin. **B.** 1×1 µm^2^ AFM height contrast image of a mixed population of spheroid oligomers and longer protofibrils. Black arrows point out examples of spheroid oligomers with various shapes and sizes. **Inset**, magnified view of a protofibril in which the underlying periodic structure is probably helical. **C.** Topographical molecular volume histogram of 341 (*n*) spheroid TTR oligomers. The numbers above the modes correspond to the mean values of gaussian fits.

TTR annular oligomers were observed to be unstable, and at some point they were replaced by other oligomerization forms, hereby collectively called spheroid oligomers, as well as protofibrils (Figure 3A-B). The stability of annular oligomers varied somewhat across different sample preparations, but as early as from 12 h after incubation they disappeared. In all cases, and within 48 h, they were replaced by oligomers with an apparently more compact and spherical shape. Spheroid oligomers are a morphologically heterogeneous population with topographical height distributed in the same range as that of annular oligomers (Figure S2), with a distinct peak at ∼1 nm and a broader peak at ∼2.8 nm. In some cases a central topographical depression, possibly reminiscent of annular oligomers, could still be observed in spheroid oligomers (Figure 3.A inset). The molecular volume distribution of spheroid oligomers displayed multimodal distribution with peaks at 55, 195, 315 and 415 nm^3^ (Figure 3.C). Transition between annular oligomers and spheroid oligomers or protofibrils was difficult to capture. However, occasionally we could observe the linkage and fusion of annular oligomers into higher-order structures (Figure 3E). High-resolution AFM analysis of such structures revealed the emergence of a helix (Figure 3F).

To monitor the transition between annular and spheroid oligomers we undertook dynamic light scattering (DLS) measurements which we coupled to AFM imaging at given time points of incubation (Figure 2). The native TTR exhibited an apparent hydrodynamic radius (Rh_app_) of 3.1 nm consistent with its tetrameric assembly (Figures 2A-B). Within two hours of incubation, the average particle size was reduced to 1.9 nm indicating the disassembly of the quaternary structure of TTR under acidic conditions (Figures 2A-B). From here onwards, signs of aggregation were initially manifested by the presence of particles with an Rh_app_ of ∼3.5 nm (Figure 2B). After 7h of incubation, larger oligomeric forms with an Rh_app_ of 15 nm appeared. With time, these larger oligomers will become more predominant (Figure 2A), but from 7h to 12 h their size showed a shortening trend that by 12 h of incubation resulted in the observation of particles with an Rh_app_ of 8.3 nm (Figure 2B). This trend is eventually reverted towards the appearance of particles with increased Rh_app_ of ∼30 nm after one week of incubation, while at the same time particles with an Rh_app_ of 8–9 nm remained persistent. In the early hours of aggregation, given the presence of smaller oligomers in larger numbers, the weighted average of particles will be less sensitive to the presence of larger oligomers, and will mostly reveal the aggregation tendency of the sample (Figure 2B). It should be noted here that, the importance of DLS measurements as performed in such system is relevant towards definition of trends in Rh_app_ rather than in the actual Rh_app_ values themselves, which due to various reasons that will be discussed below, can only be taken as an approximate value. Consistent with the trends observed by DLS, AFM imaging in liquid of the sample after 9 h of incubation when it showed the presence of larger oligomers with an Rh_app_ of 13.7 nm revealed the presence of annular oligomers with a diameter varying beteen 12 and 16 nm (Figure 2C); but at 12 h they were not observed anymore, and only spheroid oligomers with heights distributed between 2.3 and 2.8 were present (Figure 2D).

Formation and growth of protofibrils was accompanied by a decrease in the number of isolated spheroid oligomers observed, indicating that assembly of protofibrils can occur at the expense of oligomers. Protofibrils with a nodular substructure (Figure 3A) continued to grow in lengths up to ∼300 nm beyond 48 hours of incubation. They displayed an axial periodicity of 17 nm [15] and an average topographical height at the top of each node of 3.6 nm (±0.7 nm S.D., n = 267; 55 particles). These protofibrils showed the tendency to bundle together. At later time points, bundles grew in size and isolated protofibrils became scarce, suggesting a reduction in the rate of protofibril formation.

### Disassembly of Amyloid Protofibrils Results in the Appearance of Distinct Annular Oligomers

**Figure 4:**
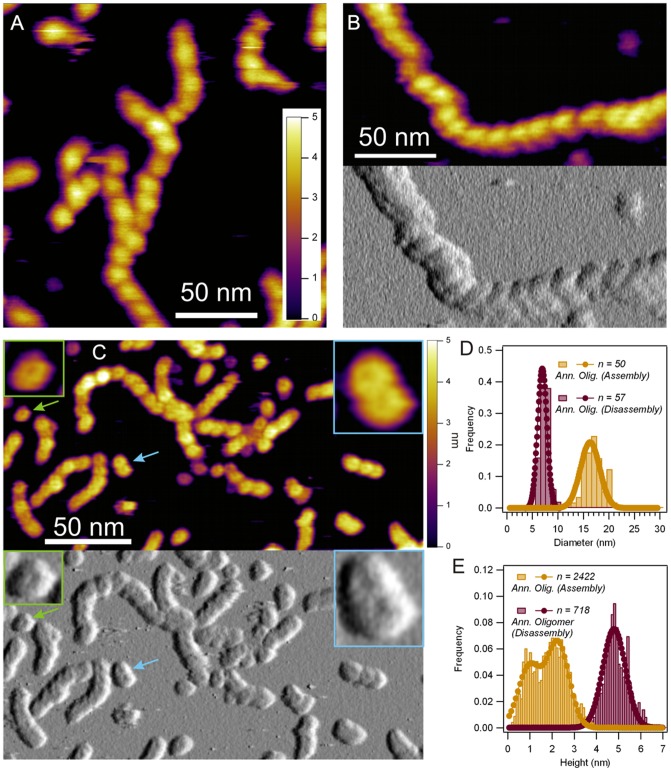
Disassembly of TTR protofibrils. **A.** AFM height contrast image recorded after 1 minute of sample dilution into PBS. **B.** AFM height (top) and amplitude (bottom) contrast images recorded after 5 minutes of sample dilution into PBS. **C.** AFM height (top) and amplitude (bottom) contrast images recorded after 15 minutes of sample dilution into PBS. **Insets**, magnified image of a single annular oligomer (left) and a laterally-associated doublet of annular oligomers (right). **D.** Distribution of the diameter of annular oligomers observed during assembly (yellow) and disassembly (purple). **E.** Distribution of the topographical height of annular oligomers observed during assembly (yellow) and disassembly (purple).

The growth of TTR protofibrils was halted by placing the sample in PBS (pH 7.4). Structural changes were detected already after one minute (Figure 4A). The topographical height of protofibrils increased to 4.8 nm (±0.5 nm S.D., n = 243 particles) while they maintained their axially periodic structure. After five minutes of incubation (Figure 4B) a further loosening of the protofibrillar structure was observed. By 15 minutes the protofibrils began decomposing into annular oligomers (Figure 4C). These oligomers displayed a uniform morphology but markedly different from annular oligomers in the assembly pathway. Notably, they exhibited a diameter of 7 nm (±1 nm S.D., n = 70 particles, Figure 4D) and mean topographical height of 4.8 nm (±0.6 nm S.D.; n = 70 particles, Figure 4E).

## Discussion

In the present work we investigated the assembly and disassembly mechanisms of transthyretin (TTR) amyloid protofibrils formed in mildly acidic conditions by using high-resolution *in situ* AFM. The AFM images of WT TTR at physiological pH, obtained at a concentration of 50 nM of TTR monomer, showed the predominant presence of particles with a calculated mean volume of 25 nm^3^ (Figure S3) and a mean height of ∼1.0 nm (Figure S2). Most likely these particles represent the monomeric and not the tetrameric form of TTR, which is known to disassemble into its constituent monomers below micromolar concentrations [17]. Indeed, using the molecular weight to determine protein molecular volume [18], [19], [20] we expect the TTR monomer (14 kDa) to be 28 nm^3^, in good agreement with its crystal-structure dimensions of 4.5×3×2 nm^3^ [21] and thus validating the AFM-based volume measurements.

### TTR Assembly Pathway

Acidification was used to evoke amyloidogenic structural changes in WT TTR and to maintain a commitment toward the amyloid-formation pathway [15], [16]. It is known that under acidic conditions the TTR tetramer disassembles into monomers that display an aggregation-prone conformation [14]. Indeed, within the first hours after acidification the sample was mostly populated by particles with ∼1 nm in height (Figure S2) strongly suggesting the presence of TTR monomers At this stage, particles show a shift of molecular volume to ∼40 nm^3^ (Figure S3) suggesting the appearance of an unresolved dimer population. These observations are consistent with DLS measurements showing a decrease in the particles Rh_app_ from 3.1 to 1,9 nm within 2 hours after acidification (Figures 2A-B).

In the next 9 hours, and in some preparations up to 48 h of incubation, annular oligomers with a diameter of ∼16 nm appeared (Figure 1). For comparison, in amyloidogenic systems other than TTR, annular oligomers with a diameter in the range of 4–45 nm have been reported [22]. The uniform circular shape and the narrow diameter-distribution of the annular oligomers seen here suggest that they represent a distinct structural state of aggregated TTR. High-resolution topographic analysis of annuli revealed an octameric symmetry (Figure 1C) where each unit is separated by 6 nm (Figure 1F). This spacing is consistent with the dimensions of a single WT TTR monomer (4.5×3×2 nm) [21] which, under acidic conditions, retains only half of its native fold, with the region corresponding to the CBEF β-sheet remaining highly unstructured [23]. Thus, in its most basic architecture, each TTR annulus is likely to contain eight partially unfolded TTR monomers. The octameric arrangement probably represents a toxic form, as it has been proposed that oligomers containing up to eight TTR monomers, but not more, are cytotoxic [24]. Incidentally, in other amyloidogenic systems such as α-synuclein [7] or prions [25], an octameric arrangement of annular oligomers has been proposed. Although annular oligomers have a uniform diameter of ∼16 nm (Figure 1D), they show some heterogeneity in their thickness. The bimodal distribution of the annular oligomer heights with peaks at ∼1 and ∼2 nm suggests the presence of at least two species: (a) a fundamental assembly containing only 8 monomers (singlet) and; (2) a double-stack of singlets (doublets) with 16 monomers. A related type of mechanism, whereby annular oligomers might stack via hydrophobic forces that induce water exclusion has been proposed previously [26]. In the case of the TTR annuli seen here the topographic height fluctuates between 1 and 2 nm along an individual annulus (Figure 1D), indicating that doublets form not by the superposition of preassembled singlets but by a gradual addition of subunits onto a pre-existing annulus that serves as a scaffold. Therefore, the population of annular oligomers is not restricted to singlets and doublets, but will likely encompass intermediate states containing between 8 and 16 monomers. Although stacks of TTR annuli may form, it is unlikely that protofibrils arise from a continuation of this process because of morphological discrepancies: whereas a TTR annular oligomer is ∼16 nm in diameter (Figure 1D), the thickness of a protofibril is only 3.6 nm (Figure S2). Annular oligomers are usually reported either as isolated entities [7], [27], [28], [29], [30] or within an overcrowded environment that makes difficult to discern any intrinsic associative propensity between them [28], [29], [31]. As seen in Figures 1A-B, annular oligomers also display a tendency to associate laterally and from short longitudinal structures. This property is not exclusive of TTR annular oligomers [32], [33]. The exact mechanisms of ring interactions are unclear. However, the lateral association and filament-forming propensity of annular oligomers observed here may point at an important step in the formation of protofibrils.

Annular oligomers appear to be a transient intermediate along the protofibrillogenesis pathway, as they disappear within 12 to 48 h of incubation and become replaced by a heterogeneous population of spheroid oligomers and short protofibrils (Figs. 3A-B). These particles generally did not display annular shape; although a subtle central depression in spheroid oligomers could sometimes be discerned suggesting that they may stem from annular oligomers (Figure 3A inset). There has been some speculation about the possibility that annular oligomers and spheroid oligomers may be part of two independent, parallel aggregation pathways [32], but it cannot be ruled out that annular and spheroid oligomers represent different conformational states of oligomers along the same pathway [34]. To clarify this possibility, we carried out molecular volume calculations of spheroid oligomers based on AFM images [15], [18], [19], [20]. The volume histogram of spheroid oligomers is dominated by peaks at 55, 195, 315 and 415 nm^3^ (Figure 3C) corresponding to dimers, octamers, dodecamers and hexadecamers, respectively. Because annular oligomers may contain between 8 (singlet annulus) and 16 (complete annular doublet) units (Figures. 1C-E), it is conceivable that the octamer, dodecamer and hexadecamer spheroid oligomers arise from structural reorganization of annular oligomers (Figure 4). This possibility is supported by recent reports indicating that amyloid oligomers display a certain degree of plasticity that allows them to convert into species of increased stability [35]. It has been observed in single-molecule-fluorescence studies on the amyloid aggregation of the SH3 domain of PI3 kinase that although the aggregation number of amyloid oligomers can remain essentially unchanged as a function of time, internal conformational reorganization of oligomers result in more stable species that would be involved in continuing the aggregation process [35]. Similarly, spheroid oligomers reported here are more persistent than annular oligomers, suggesting that they are more stable.

Although the mechanisms of the structural transition involving annular oligomers are unclear, AFM images of associating annular oligomers (Figure 2F), particularly the ones recorded in amplitude- and phase-contrast mode, are suggestive of a ring-to-helix transition. Such conversion would likely involve the spatial rearrangement of the structural units that compose the annular oligomers providing a more compact conformation that would minimize the surface exposure of hydrophobic areas to water as suggested earlier for Aβ1-42 oligomers [36]. If such possibility is indeed plausible, it is expectable to observe changes in their hydrodynamic radii. To investigate this possibility we followed the aggregation process by DLS since the initial 14 h, and later after 24 h and past 7 days since the start of aggregation (Figure 3A and 3B). During protein aggregation, the varying shape of oligomers and the high polydispersity of the sample will impose strong limits to the accurateness of DLS measurements. However, monitoring the overall changes in the apparent hydrodynamic radius (Rh_app_) of particles undergoing aggregation bears relevance to understanding the dynamics of such systems. To further elucidate any changes in particle size, at certain time points the sample was imaged by AFM in liquid (Figure 3C) to help to capture the moment of formation and evolution of annular oligomers. The initial two hours of aggregation were dominated by dissociation of TTR tetramers, as seen by the decrease in the Rh_app_ from 3.1 to 1.9 nm (Figure 2A-B). After 2 h of incubation, particles with a Rh_app_ of ∼3.5 nm emerged and 7 h after incubation a population of larger oligomers appeared. These larger oligomers, initially with an Rhapp of ∼15 nm will, after a further 5 h of incubation, have their Rh_app_ decreased to ∼8 nm. Consistent with the shortening in Rh_app_ the AFM images recorded at 9 h and 12 h of incubation (Figures 2C-D) show marked differences, with annular structures being observed at 9 h and more compact spheroid oligomers being dominant at 12 h. Thus, although the dynamics of transition involving annular oligomers may in fact be more rapid and complex than what we were able to capture, it seems possible that annular oligomers may form and later collapse in a series of events that can take approximately 6 h.

Concomitant to spheroid oligomers, short protofibrils were also present in the sample (Figures 3A-B). As a function of incubation time, larger spheroid oligomers disappeared and only protofibrils and a small population of monomers/dimers remained as it is also suggested by DLS measurements where after 7 days of incubation where particles with an Rh_app_ smaller than that of the TTR tetramer are observed. Thus, either the larger oligomers dissociated into monomers/dimers, which would then be incorporated into protofibrils, or they directly contribute to protofibril growth. Which type of polymerization mechanism underlies the formation of amyloid protofibrils remains an open question. However, in figure 3A (inset) spheroid oligomers with morphological features reminiscent of annular oligomers can be observed to align, forming a linear structure very similar to a typical protofibril shown in figure 3B (inset). This suggests that, at least in the first days of aggregation, association on spheroid oligomers into protofibrils might be the dominant mechanism of protofibril formation and growth as it has been proposed in other cases [17], [36], [37], [38], [39], [40], [41], [42]. However, we cannot rule out that protofibrils can also arise directly via the fusion of annular oligomers followed by structural transitions (Figures. 2E-F), particularly during the initial phase of protofibrillogenesis where spheroid oligomers are absent.

### Protofibril Disassembly Pathway

Previous reports have shown that acid-induced amyloid aggregates can depolymerize upon exposure to neutral pH [43]. Recently we have demonstrated that exposure of TTR protofibrils formed in acidic conditions, as reported here, to physiological buffer conditions resulted in the rapid disassembly of protofibril bundles into component protofibrils with increased thickness and a noticeable axial compaction apparent from the reduction in their periodicity from 17 to 12 nm [15]. Here we report that annular oligomers emerge not only during the protofibril assembly pathway, but during the disassembly pathway as well (Figure 4). The radial expansion of the protofibril is evident already after a one-minute incubation in PBS (Figure 4A). After a five-minute exposure to physiological pH the protofibril structure acquires a more loosened structure. The change is particularly evident in the amplitude-contrast AFM image (Figure 4B). This later image also reveals that protofibrils contain a substructure which is more complex than a simple chain of monomers. These substructural details - for example the type of helicity, if any – remain elusive. But the dimensions and periodicity of these protofibrils and the fact that they are not a simple chain of monomers is in agreement with the double helical model of the TTR protofilament proposed by Blake and Serpell [44]. Incubation for 15 minutes resulted in the dissociation of the protofibrils into annular oligomers (Figure 4C). Although both the size and shape of these oligomers were uniform, they were different from those seen during TTR assembly (Figure 1). Oligomer diameter nearly halved from 16 to 7 nm (Figure 4D), while the topographical height increased substantially and its distribution became monomodal (Figure 4E). Considering the large differences between the assembly- versus disassembly-pathway oligomers, it is unlikely that the latter arise simply due to the perturbation of the equilibrium constants between oligomeric states of the assembly pathway. Rather, protofibril disassembly in PBS constitutes a distinct pathway where additional structural transitions within the protofibril need to be invoked. The final fate of these annular oligomers is currently not known but is likely to depend on the various factors that typically govern the amyloid aggregation process and the molecular details of the aggregated protein itself. For example, it has been shown that phosphoglycerate kinase in the form of amyloid-like fibrils, generated by incubation at pH 2 for 7 days, is able to recover over half of its activity at neutral pH conditions [45]; WT TTR can aggregate into amyloid-like fibrils in PBS buffer once the protein has been subjected to acidic pre-treatment for 3 days [46]. Thus, the disassembly-pathway annular oligomers observed here may still serve as assembly blocks for another form of amyloid-aggregation.

### Protofibrillogenesis Model

**Figure 5:**
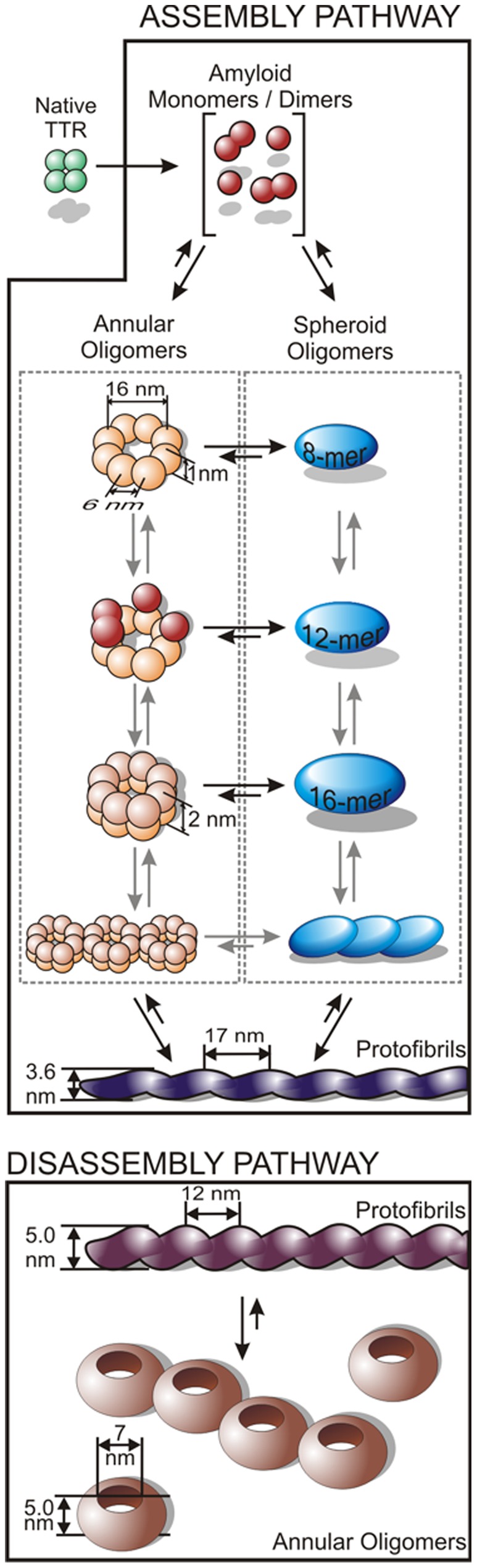
Model of TTR protofibril assembly and disassembly. Relevant dimensions and periodicity parameters of the intermediates are indicated where applicable. Length of the arrows scale with the hypothesized transition kinetics.

Based on our findings we propose the following model for the assembly and disassembly of TTR protofibrils (Figure 5). Upon acidification, native TTR undergoes structural transitions [16], [47], and the tetramers dissociate into amyloidogenic subunits (predominantly monomers and, in smaller quantities, dimers) from which annular oligomers with octameric symmetry assemble. A single annular oligomer may serve as a scaffold for the continuous addition subunits up to the formation of an annular doublet containing 16 monomers, but further stacking appears unlikely. Rather, the annular TTR oligomers may associate laterally to form the first linear aggregates observed, but are even more likely to structurally reorganize into spheroid oligomers containing between 8 and 16 units. Spheroid oligomers coalesce in a dynamic equilibrium with the growing protofibrils which are abundant in the first week of incubation. Upon adjusting pH to neutral, protofibril structure quickly reorganizes, and dissociation into an annular oligomeric species quite distinct from that seen in the assembly pathway proceeds on the minute time scale.

### Conclusions

Annular oligomers have been reported to appear in a variety of amyloidogenic preparations and proposed to form cytotoxic membrane pores [7], [22], [25], [27], [28], [29], [30], [32], [33], [48], [49], [50], [51], [52]. Here we have demonstrated that TTR, which is well recognized in the etiology of certain types of amyloidoses, also forms annular oligomers. TTR annular oligomers are apparently not off-pathway structural states but intermediates of the protofibril assembly. They appear to form and collapse in a time scale of a few hours resulting in the formation of more compact spheroid oligomers which will be the main dominant oligomeric species during initial stages of protofibril growth. The fact that protofibrils themselves disassemble via formation of annular oligomers further suggests that the two types of oligomeric arrangements are indeed related. The exact consequences of these structural changes towards cytotoxicity are beyond the scope of this report; however, it is an interesting possibility that if indeed toxicity arises from annular oligomers, it may depend on a delicate balance between annular and spheroid/protofibril states, as an opened and closed state, which may be tuned by local environment.

## Materials and Methods

### Sample Preparation

Recombinant WT TTR was prepared as described previously [53] and isolated to high purity by using anion exchange (MonoQ column, GE Healthcare) and size exclusion (Superdex S75 column, GE Healthcare) chromatography steps. Stock solutions of WT TTR were kept in 10 mM HEPES, pH 7.0 at −20°C. Protein quantification was performed by spectrophotometry (ε_280_ = 77600 M^−1^ cm^−1^). TTR fibril formation was induced by diluting TTR to a final concentration of 1 mg/mL in 50 mM sodium acetate buffer at pH 3.6 and incubating at 37°C [15]. Typically, maturing TTR samples were monitored for up to two weeks. TTR protofibril disassembly was initiated by adding a 2 µL protofibril sample to 50 µL of phosphate buffered saline (PBS, pH 7.4). Disassembling TTR protofibril samples were incubated at room temperature for 1, 5 and 15 minutes. For AFM analysis, the samples were diluted by adding 948 µL ultrapure water.

### Atomic Force Microscopy

All images were acquired in liquid with an MFP-3D AFM instrument (Asylum Research, Santa Barbara, CA) using a cantilever (Biolever A, Olympus) with a spring constant of ∼30 pN/nm and a resonance frequency of ∼9.2 kHz. Native WT TTR was imaged at a concentration of 1 µg/mL (50 nM of monomer) in 50 mM HEPES (pH 7) buffer containing 150 mM NaCl. Aggregated samples were diluted 500× prior to deposition on freshly cleaved mica. Non-contact (AC) mode AFM images were acquired using free and set-point amplitudes of ∼0.3 V and ∼0.2 V, respectively. Images of 1024×512 and 512×512 pixels were obtained at a scanning frequency of ∼0.8 Hz.

### Dynamic Light Scattering

DLS measurements were carried out by irradiating the sample using a Melles Griot diode-pumped solid-state laser (457.5 nm) and scattering data collected at 90°. Autocorrelation functions were calculated by a in house developed acquisition system and the particle size distributions were determined by the maximum entropy method [54] assuming the presence of spherical particles. To eliminate overestimation of very large aggregates from the scattering intensity signal, the results were analyzed between 1 and 100 nm and by plotting the fraction of scattering volume as a function of hydrodynamic radius.

### Image Processing and Data Analysis

Images were processed by using standard procedures in IgorPro v6.04 (Wavemetrics) and user procedures developed by Asylum Research as part of the MFP-3D software package. Molecular volume calculations based on AFM images were performed as schematically illustrated in the Figure S1 and in accordance with previous reports [15], [18], [19], [20].

## Supporting Information

Figure S1 **Schematic representation of the effect of tip geometry in the observed particle size, leading to an apparent increase in width.** To mitigate this broadening effect, particle volume calculations of spherical particles were made using their apparent diameter (d) at half-height (h/2) together with the particles’ maximal height (h) as indicated in the materials and methods section.
(TIF)

Figure S2 **Height distribution of different particles as indicated in each plot.** In all cases, the data was fit to Gaussian functions.
(TIF)

Figure S3 **Molecular volume distribution of different particles as indicated in each plot, and for which a spherical geometry was apparent.** In all cases, the data was fit to Gaussian functions.
(TIF)

Figure S4 **1×1 and 5×5 µm^2^ scans in AC mode on air after drying a sample containing annular oligomers.** No annuli can be observed and instead only spherical particles, with heights of 10 to 15 nm can e observed and indicating that the drying process greatly interferes with the morphology of particles.
(TIF)
