# Supplementary figures and images for: Distinct Annular Oligomers Captured along the Assembly and Disassembly Pathways of Transthyretin Amyloid Protofibrils
Source: PLoS One. 2012 Sep 12;7(9):e44992. doi: 10.1371/journal.pone.0044992 (PMC3440338; doi:10.1371/journal.pone.0044992)

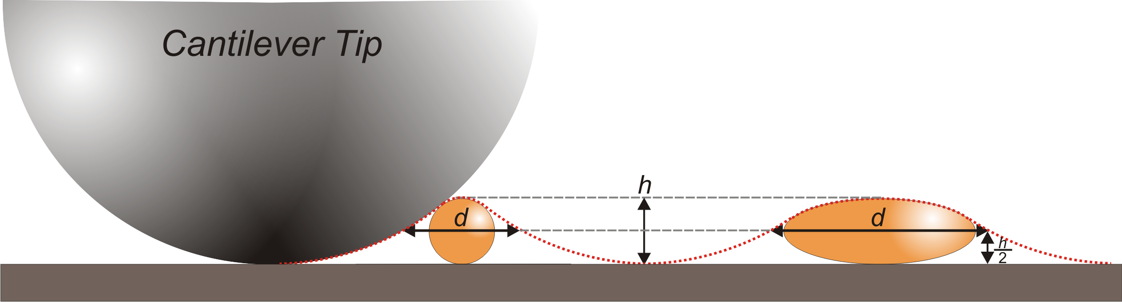

Supplement: Figure S1 — Schematic representation of the effect of tip geometry in the observed particle size, leading to an apparent increase in width. To mitigate this broadening effect, particle volume calculations of spherical particles were made using their apparent diameter (d) at half-height (h/2) together with the particles’ maximal height (h) as indicated in the materials and methods section. (TIF) [file pone.0044992.s001.tif]

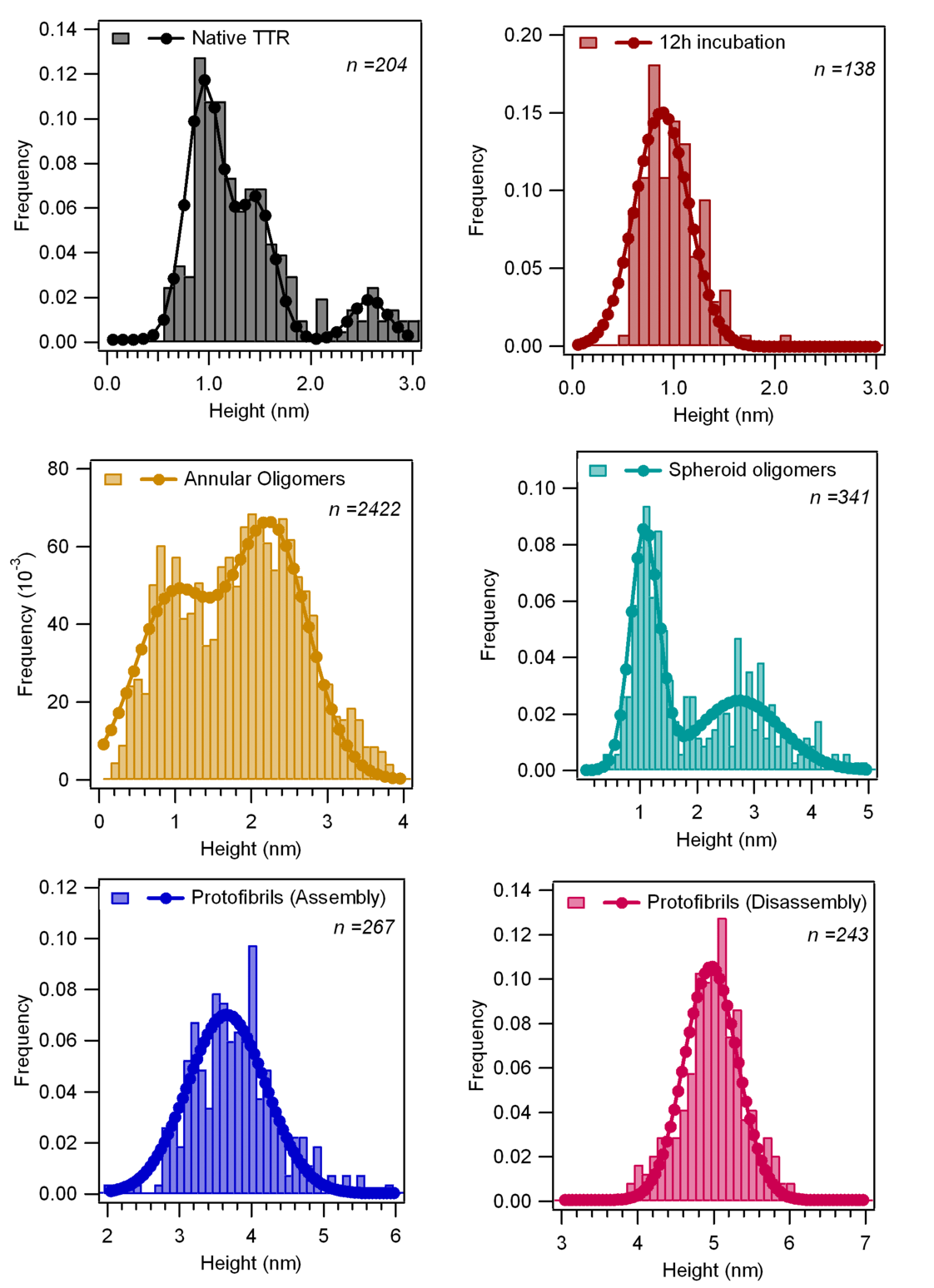

Supplement: Figure S2 — Height distribution of different particles as indicated in each plot. In all cases, the data was fit to Gaussian functions. (TIF) [file pone.0044992.s002.tif]

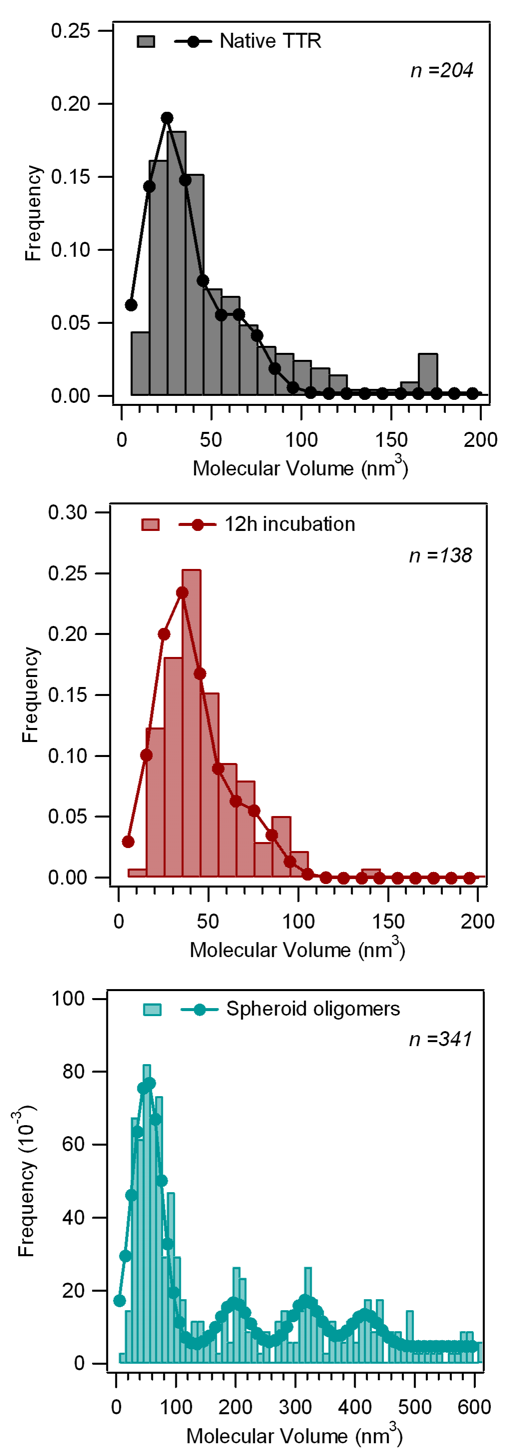

Supplement: Figure S3 — Molecular volume distribution of different particles as indicated in each plot, and for which a spherical geometry was apparent. In all cases, the data was fit to Gaussian functions. (TIF) [file pone.0044992.s003.tif]

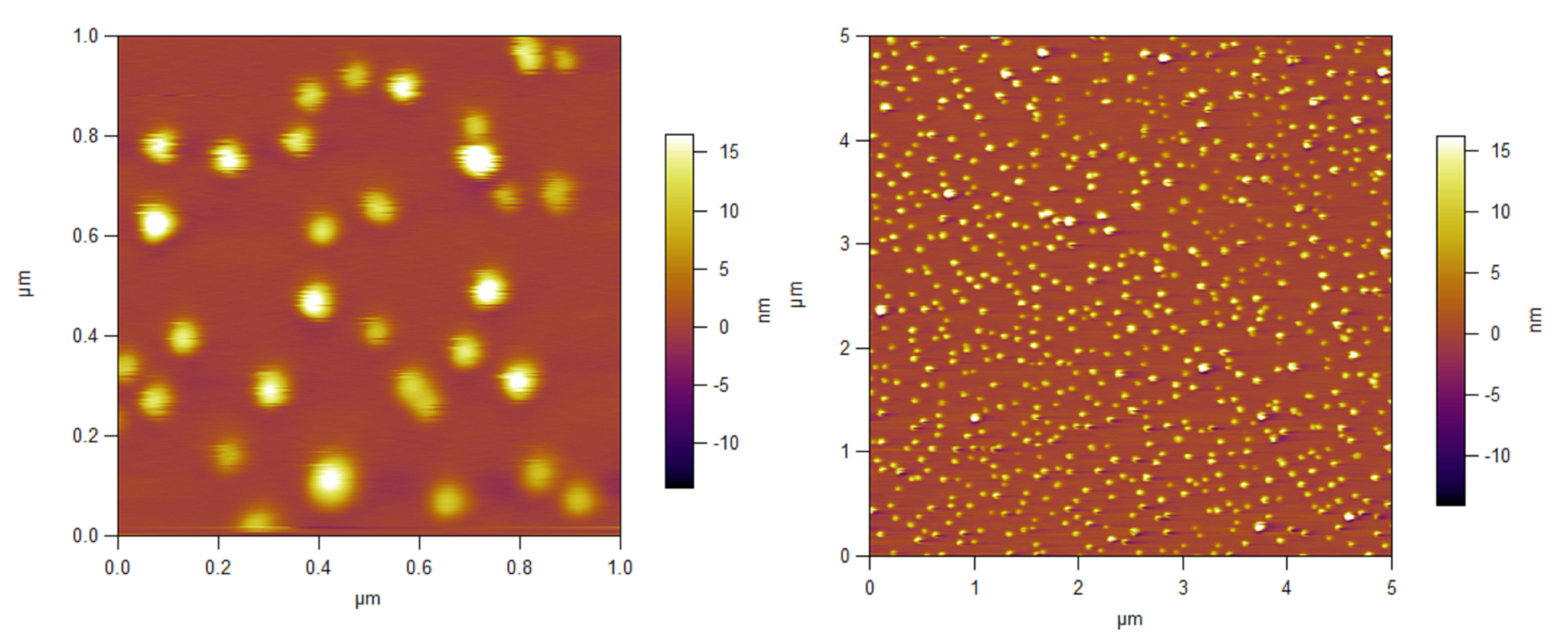

Supplement: Figure S4 — 1×1 and 5×5 µm2 scans in AC mode on air after drying a sample containing annular oligomers. No annuli can be observed and instead only spherical particles, with heights of 10 to 15 nm can e observed and indicating that the drying process greatly interferes with the morphology of particles. (TIF) [file pone.0044992.s004.tif]
